# Parents' Attitudes to Risk and Injury to Children and Young People on Farms

**DOI:** 10.1371/journal.pone.0158368

**Published:** 2016-06-30

**Authors:** Kerstin Nilsson

**Affiliations:** 1 Department of Work Science, Economic & Environmental Psychology, Swedish University of Agricultural Sciences, Alnarp, Sweden; 2 Division of Occupational and Environmental Medicine, Lund University, Lund, Sweden; Institute for Health & the Environment, UNITED STATES

## Abstract

**Objectives:**

Children and young people growing up in a farm environment run a greater risk of being injured or dying in an accident than their non-farming counterparts. This study examines farming parents’ attitudes and experiences of having their children grow up on farms, one of the most dangerous work environments as their home, everyday environment and playground.

**Method:**

Data were collected using two ethnological methods, a question list and interviews, with a study population of 20 parents. The data were analysed phenomenologically.

**Results:**

The analysis pursued four themes: i) the most dangerous places and situations on the farm; ii) children’s tasks on the farm; iii) children as a safety risk on the farm; and iv) farm risk education for children.

**Conclusions:**

Most parents know the risks on their farm, but are sometimes careless when working under stress or exhaustion. Some parents wanted more information and some wanted compulsory preventative or safety measures by manufacturers, e.g. a safety belt as standard on the extra seat in tractors. Children’s friends were described as one of the greatest risks for injury due to peer pressure. Some parents mentioned that people who grow up on farms are sometimes ‘blind’ to the dangers. Other parents seemed to overlook the risks and had their children carrying out tasks for which they were not mentally or physically equipped. Some of the tasks the children reportedly carried out on farms contravened Swedish legislation. It is thus important for farming parents to be repeatedly reminded of the risks to their children and to increase their awareness of how to prevent and eliminate risks in order to avoid accidents on the farm. The situation for farm children is highlighted in a critical discussion.

## Introduction

Agriculture is one of the most dangerous work environments, with many injuries and high mortality [1–6]. Reviews on interventions in agriculture stated the lack of optimally injury preventions in agriculture [7–9]. At the same time, farms are the residential and play environment for some children. Children and young people who live, move around and play in this exposed environment run a greater risk of being injured [7;8;10–18]. People’s behaviour often depends on their earlier experiences and learning to make the same reactions as before in a given situation [19]. Children often imitate the behaviour of adults and other children. That behaviour can be changed in a healthy or unhealthy direction, by positive reinforcement and modelling [20–23]. Risk-taking behaviour among both males and females is often modelled on influential others in the environment and their behaviour during childhood [24].

Accidents are still the most common cause of death among children [7;12–18; 25]. In United States (US) 45 children are injured at farms every day, and every three days a child dies in accidents related to agriculture [14]. Nearly half of all fatal injuries among youth under the age of 17 in the US occurred in agriculture [13]. There are currently still too many children injured, mutilated or killed in accidents, and children living on farms are at higher risk than hired workers.

In many countries in the western world the fair labour standard acts established a minimum age of 16 years for non-agricultural employment [13]. There are most often restrictions for youths in order to keep them from the most hazardous tasks. However, the child labour restrictions in some countries exclude children of farm owners or farm operators. In Sweden the laws concerning the work environment [26] and young people at work [27] do not allow children under 13 years of age to work with any tasks other than picking fruit and berries, easy feeding and weeding without a permit. At the age of 16, young people may take on normal, non-dangerous work, but have to be over 18 to work with animals, tractors, farm machinery, machine-powered tools and equipment, lifters and winches. Despite this, children perform tasks that are not allowed, e.g. children driving tractors on public roads is sometimes mentioned in police reports on incidents and injuries [28].

Previous studies stated lack of research regarding farming parents’ attitudes towards their children’s risk at farms and towards regulatory approaches that specifically target children [7]. Therefore, the main aim of this study was to examine parents’ attitudes and experiences of having their children grow up, play and work on farms, one of the most dangerous work environments.

### Background

Farm businesses are often owned by families and husbands, wives, children and grandparents often participate to some degree in tasks on the farm [12–18]. The interaction between the family farm business and family members can be complicated [29;30]. Family members often feel an expectation to be loyal to the business. Family relationships, family traditions, culture and feelings have a great influence on the business. While the economic situation in the business, the company values, the working hours and the business objectives have a great influence on the family. Family businesses often give the children the benefit of taking responsibility at an early age, but this can be a hinder to their unique individual development. If a family member is always expected to act to satisfy the interests of the business, it may be almost impossible to break the tradition of respect to the family and family solidarity [29;30]. Many farm businesses are handed down from parents to children and this could make it more difficult to decrease the risks on the farm without creating conflict between family members. Growing up in the family farm business can sometimes make it more difficult to observe possible safety risks in the environment [12–18;30]. People who run a farm business in which they have grown up are sometimes blind to the dangers and cannot perceive safety risks. In addition, a human defence mechanism in managing a life situation and conditions that are impossible to change is to simply deny unpleasant events and threats [31;32]. Individuals who live and visit places with many risks and dangers often ignore these in a daily situation [33].

Family members are at risk on farms through living in a workplace and through participating in daily tasks, and 32% of reported accidents on farms involve a family member [34]. Many of the children who die in accidents live on farms [14;35]. The Delegation for Children’s Safety states in its report that it is important to increase children’s and young people’s development and safety in the community [36]. This should be done by removing risks to their health and wellbeing, empowering them to visit and play in the surrounding environment. At farms, non-fatal injuries are most common among boys aged 10–15, and pre-school children and male adolescent have a higher risk for fatal injury [14]. A study concluded that in accidents to farm children, girls are most commonly injured by horses and boys by machinery [37]. A Norwegian study found that 60% of accidents to children on farms occurred during children’s play and that the most damaging and fatal accidents involved tractors, machines and tools [38]. The study also noted that 25% of the injured children were visitors to the farm. Statistics from the USA show that of the children and adolescents injured on farms during 2001, approximately 32% were under the age of 10, about 49% were aged 10–15 and 19% were aged 16–19 [39]. Children aged six years or below often take unexpected actions that increase the risk of accidents, especially in dangerous environments [40]. On the other hand, adolescents are more often allowed to visit dangerous places and situations on the farm and they are more commonly injured when the environment acts unexpectedly. Roughly half of all injured children are under surveillance by an adult who, at the same time, is actively conducting farm work [14]. Another study found that two-thirds of children killed in farm accidents were accompanied by an adult, and that in half these accidents the adult was close to the child [41]. In one-third, the adult had given exact instructions and information to the child before the accident. Parents often overestimate their own children’s skills and abilities [42].

Engineering controls have good potential to prevent farm injuries, but only if they are voluntarily adopted by the farmers [7]. There is a lack of work standards on farms which regards to the farmers’ value of independence and self-sufficiency. However, systematic work has been done in recent years in order to protect children from the risk of injury. One is the rollover protection structure (ROPS) on farm tractors. In Scandinavia the tractor rollover-related deaths has been reduced to null [43]. In North America, ROPS has been voluntarily introduced as standard equipment on new tractors. However, a lot of farm children in many countries are still operating tractors not equipped with ROPS [44]. Despite recommendations to promote agricultural safety and health towards children, the adoption of preventive strategies is still not widespread [7;12–18] In an attempt to reduce accidents to children on farms, in view of the studies cited it is relevant to examine the views of farming parents to their children’s situation in their home environment.

## Material and Methods

Narratives were obtained from parents about how they viewed their child's everyday living and growing up on a farm, one of the most accident-affected workplaces. Efforts were made to bring in stories from across Sweden in order to obtain a geographical spread of farms. A mixture of two ethnological methods, a question list and semi structured interviews, was used, as this is reported to be an optimal and well-known combination in other studies [45].

The question list method has been used by anthropologists and ethnologists in Sweden since the early decades of the 1900s [43,45]. It is a data collection method in which individuals get inspiration, ideas and instructions to share their own views, memories, experiences and values from a list or letter with the theme and issues. The data collected are therefore autobiographical, because there is no intermediary, for example an interviewer. The narrative writer decides how and what should be highlighted in the stories produced. Therefore the researcher does not influence the reported narrative as much as in an interview situation. However, a disadvantage of the question list method is that individuals may experience the method as "vague" and find it difficult to get going and really know what the researcher is looking for, or misunderstand the purpose. Some may feel that it is difficult to express what they want to say in writing, or are poor at spelling and therefore reluctant to write down their stories.

### Study population

To reach parents of children in agriculture, the question list was conveyed in three ways: 1) It was published in the farm journal *Land Lantbruk* (Country Farm). 2) It was sent together with a covering letter by email to farmers affiliated to the Federation of Swedish Farmers (LRF) through LRF regional membership lists. 3) It was presented with an oral request at an LRF Youth Meeting. After three months, the story collection period was terminated. A total of eleven stories were collected and all very informative, with some respondents even having illustrated their narratives with photographs.

Because the question list method only received eleven responses, it was decided to supplement the data with interviews to achieve information saturation of the objectives. This is also reported to be an optimal and well-known combination in other studies [45]. Researchers commonly use interviews as a supplement and together with a question list for the collection of materials and knowledge about a topic [45;46]. The LRF municipal chairman and snowball sampling produced the names of farming families and ten farming parents agreed to participate. These were contacted and an appointment was made for semi-structured interviews by telephone. A telephone interview is more anonymous than a face to face interview, and the researcher cannot read the informants’ body language. However, it could be easier for the informant to express sensitive things, because they are hidden and secure by themselves in their home and not exposed to anyone who observes them.

In total, ten semi-structured telephone interviews were held with farming parents geographically distributed throughout Sweden. In the interviews the question list was used and reconstructed to a semi structured interview guide, and it was therefore possible to combine the results from the stories and the interviews in the subsequent analysis. However, the questions in the written question list have a broader description with many small follow up questions to get a broader perspective to reflect on, to choose from and respond to for each of the three head questions. Those follow up questions was also used in the telephone interview to probe deeper in the respondents’ reflections. The head questions in the question list and in the semi structured interview guide were: Describe your child/children and adolescents activities and tasks at the farm; Describe risks for accidents and injuries at your farm; Describe how you and other adults at the farm manage your child/children and adolescents risk of injuries.

After 16 collected narratives (ten from the question list and six interviews) there was no additional new information. The answers only repeated the former collected narratives. However, to be sure of this we went on until 20 narratives were collected. After this we were convinced that there would not be any new information added from any more interviews; the data collection was saturated and completed. This is also the stated way to make sure that the data collection is powered in qualitative studies [45;46].

The ten interviews were then transcribed by the researcher. The final sample in the study consisted of 20 parents, seven men and 13 women, who together represented 50 children aged between six weeks and 17 years. Of these, two men and nine women submitted narratives based on the question list, while five women and five men participated in interviews. One of the women who participated in the interviews had also sent in a narrative. Those did not contain the same information, but additional and more detailed information from her life as a farming mother. Her narrative and interview were therefore used together in the analysis. The informant parents were spread throughout Sweden, from Umeå in the north to Trelleborg in the south. This gave satisfactory representation in the respondent group for the objectives of the study. The respondents were engaged in agricultural production specialising in e.g. beef, swine, sheep, horses, poultry, crop production and forestry. At least one of the parents was employed full-time in work on the farm in all cases except one, where one parent worked part-time on the farm.

### Analysis

The collected data were then analysed by the phenomenological method to crystallise the essence of the parents' attitudes toward their children's risk of getting injured in agriculture from the collected narratives and interviews [46]. The analysis was performed in a number of steps [45;46]. First, the texts from interviews and narratives were analysed together to identify basic ideas that reappeared and were typical of the stories. In the first step of the analysis all the text was read three times. In the second step, specifically interesting crystallised parts in the text were marked in different colours. In the third step, these different sub-themes were grouped into sub-themes and in the fourth step they were clustered into themes. Finally in the fifth step, four themes were identified. These were: The most dangerous places and situations on the farm; Children’s tasks on the farm; Children as a safety risk on the farm; and Farm risk education for children. The results are reported in the following sections based on these categories/themes. The results are then compared with previous research in a concluding discussion.

The investigation was conducted according to the principles expressed in the Declaration of Helsinki. All the participants were given written information about the study before their participation. Written informed consent was obtained by mail from the participants who sent in their narratives based on the question list. Verbal informed consent was obtained from the participants in interviews and this was recorded in the beginning of the telephone interviews. The data were analysed anonymously. The study was approved by the departmental review board at the Swedish University of Agricultural Sciences. The study was funded by The Swedish Farmers’ Foundation for Agricultural Research and was performed by the Swedish University of Agricultural Sciences, Institution of Work Science, Business Economics and Environmental Psychology, Alnarp. The funders had no role in study design, data collection and analysis, decision to publish, or preparation of the manuscript.

## Results

The results of the analysis of parents’ attitudes to risks to their children on farms are presented below based on the four themes and illustrated by quotes from the parents’ narratives.

### The most dangerous places and situations on the farm

The parents perceived an increased risk of their children being injured at times and in situations when there was too much to do on the farm and therefore a high stress level and rushed priorities. However, the parents reported that they worry every day about what could happen to their children and the risks on farms. As one mother put it: “As a mother, I am worried about my children all the time. Heavy traffic such as the feed truck, milk tanker and also our own tractor make their way across the yard and the children’s environment almost every day.” (A mother of two children).

Some of the parents had made their own risk assessment of the farm environment when they had children. One mother says: “We carried out a farm inspection on our own when the children came. We checked the drains, gates and so on. Before that, this was absolutely a very dangerous environment.” (A mother of two children).

The places on the farm that most parents perceived as dangerous for their children were close to machinery and country roads, animals, drains, ponds, hay lofts and slurry tanks. Many parents mentioned that they felt it was safer to have their children with them inside the tractor or other vehicle, so that they knew where the children were and did not run the risk of running over them with machinery, which they saw as an obvious risk. Most of the parents reported having an extra seat in the tractor, and three parents also stated that they had fitted seatbelts to this extra seat. Some parents thought that this should be standard on every tractor and vehicle. One father said: “I think it is particularly bad that a seatbelt is not standard on the passenger seat within tractors. The manufacturer should always make them like that, instead of it simply being an optional extra.” (A father of one child). Only two of the 20 parents had a seat belt for this extra seat. One parent who thought there should be a law about this said: “I think it is very poor that there is not a seat and seat belt in all tractors today. Yes, that they aren’t made like that instead of being an extra!” (A father of one child). Another father explained why he thought it should be standard to have a seat belt for the extra seat: “When the children were younger it happened that they fell asleep when they came with me in the tractor. When I hit the brake or turned I had to catch them with my elbow before they fell off the seat and crashed to the floor or into the equipment. Of course it happened that they fell down sometimes and hurt themselves. There were never serious consequences of these accidents, but it has happened.” (A father of two children).

The parents felt it was very valuable for their children’s personal development to grow up together with animals and learn to interpret the animals’ signals so they could recognise, understand and react in time. However, handling animals also brings safety risks according to the parents, because animals are not always predictable. Some parents mentioned that an animal which seemed to be very calm could suddenly react unpredictably. One mother recounted: “The quiet cow was probably frightened by the rustling sound from the two-year old boy’s winter overall. She took a giant leap, kicked and hit him in the chest. He flew through the air, hit the wall and fell to the floor.” (A mother of three children).

That child ‘only’ suffered concussion and came home from the hospital after a few days. Many of the parents reported that they talk to their children about the risks, but they cannot protect them in all situations and all the time on the farm because they have to do their daily work. The tolerance of what the children were allowed to do increased with the parents’ need to be left in peace and finish their tasks. One father described the fear he sometimes feels when he does not know where the children are when he is working on the farm: “Of course it happens that the children run away and we don’t know where they are. In these moments you run to the roads, drains and all over. The things that you are most afraid of circle around in your head while you run around and look for them.” (A father of four children).

The parents reported that long spells of work decrease their attention to safety. They try to avoid the risk of their children being injured by absolutely banning them from some places on the farm. One parent noted: “I don’t want the children to be in some places on the farm. For example the place we mix up the feed and things like that. If you have driven the tractor forward and backward for many hours then you forget to keep up the attention because you do it so many times, over and over again.” (A father of four children).

Some farms have an unsafe surrounding environment, for example with rivers, ponds and main roads around the farm. Some parents described their fear about this element that they could not change to protect their children. One father who owns a farm next to a main road said: “It is forbidden to be in the area around the main road. It is a question of life and death. The children have realized that there is no point in naming the cats, because most do not live more than two or three months due to the road. So the children have learned what will happen if they go out on the road and do not do it.” (A father of two children).

Some parents talked about the importance of orderliness on the farm and claimed that it is very important to keep things in good order so as to prevent accidents. Equipment that was broken or not properly stored could sometime be a safety risk. Other parents mentioned that farmers have problems taking help from others and that their pride could be a safety problem. One parent said: “Some farmers want to do everything by themselves, but no-one can be good at everything. It could be a safety risk if things are not in proper order and repaired in the right way.” (A father of four children).

#### Summary

The places on the farm parents perceived to be the most dangerous for their children were around traffic, animals, machines and drains, ponds, hay lofts and slurry tanks, but they pointed out that there were different risks in different age groups. The parents’ total descriptions stated that work environment areas on farms are not a good place for children to stay and play. Despite this, most parents let their children play around and stay in the food production industry areas, without any deeper reflection on not allowing them to do so.

### Children’s tasks on the farm

The farming parents pointed out the difference between their children’s life environment and that of other children. One mother said: “There are not so many who bring their children to their workplace today, whether they have a dangerous work environment or not. Our children’s home environment is at our workplace, and they have no choice about that.” (A mother of two children)

Most parents mentioned that they wanted to spend time with their children and wanted them to share in everyday life on the farm, which actually was their own work situation and tasks. One father said: “It is risky to bring him into the work, but at the same time we think it is sad if he cannot be together with us.” (A father of one child). The farming parents work many hours, and long days and one way to meet their children, but also to care for them, was to bring them in the work. One parent says: “The children have been together with us in the barn from the time they learned to walk. Before that we brought them on the baby swing hanging on the rail for the milking machine, and even before that they were with us in the barn in their pram.” (A mother of three children). Another father said: “We are positive towards child labour when it comes to keeping our children with us in the work. Our children often play work. They are with us on their own terms, for fun and as long as they want to.” (A father of three children).

Many parents thought it was important for their children to participate in tasks on the farm, and described that as a reason to bring their children to their work environment. Many parents reported that their children participate in almost every activity on the farm. It was up to the parents to be extra careful in their work tasks when the children were with them. A father said: “It is a particular risk to involve the children in the work. When I drive the tractor, for example in the spring when we move around and pick up stones from the fields, it is important that I make sure they are behind and not in front of a wheel or something. So of course there are risks when involving the children in the work.” (A father of two children).

Some parents were very proud that their children were so interested in the farm. They reported that the children’s work on the farm aroused their own interests and conditions. Some parents proudly described four-year-old children being responsible for lamb feeding. Some had children who had driven loaders and tractors since they could reach the pedals. Other parents talked about a 10-year-old who milks a couple of cows and drives the round baler compress. Others described how their 11-year old child had shovelled manure and mucked out racehorses. One mother said: “Our 11-year-old son is very interested and involved in the work on the farm. He started some years ago to drive the tractor and loader. The tasks he performs are driving the side tractor wagon when we thresh, the roller and such things.” (A mother of two children).

Not all parents described this scenario. Some mentioned that their children were not very interested in farming and did not want to participate too much. This raised ambivalent feelings according to one parent: “Our children seldom participate in the work. It is sad in one way, but at the same time they avoid dangerous elements.” (A mother of two children).

#### Summary

Most of the parents reported with pride that their children participate in work tasks on the farm. The activities and tasks that the children in the present study were involved in mostly depended on the children’s age, but not always. The parents also described that it was very dangerous to bring their children to their work at the farm and to participate in the tasks. However, they did this anyway.

### Children as a safety risk on the farm

The farming parents reported that when many children, friends or siblings are together they can encourage each other to do forbidden things. One mother pointed out that there is an increased risk when her children bring home friends to the farm: “My children one day had a group of friends here at the farm. They wanted to look cool in front of each other so they climbed up to the hay loft, even though they knew that it was forbidden. One of our girls fell down and cracked her skull. It happened so quickly. We cannot be everywhere and protect them.” (A mother of three children).

The risk with friends was perceived as particularly great when the friends are not from farms. One parent said: “I think the risk is worse when it come friends who are not familiar with farms and they run and look everywhere. Our children are used to it and they have learned about the danger. I think there are not so many risks if a single child comes, but if they come together that could be worse. That is when the dangerous situations occur.” (A father of four).

Some parents reported that they look after their children more carefully when they have friends home on the farm because of the increased risk this creates. They claimed that they did not want their own children to have the responsibility for controlling their friends in order to avoid accidents in the farm environment. One mother said: “I do not want to put the responsibility for other children on my own children if they start to nag about doing forbidden things. It is better if I as a mother forbid and rebuke instead of my child having that responsibility.” (A mother of three children).

The parents pointed out that it was not only visiting children who make their own children to do forbidden things. Their own children sometimes want to get high status by doing dangerous things. One mother says: “My son wants to show the farm to his friends when they come, and he always wants to show them the most dangerous places and things. Probably to improve and look cool in front of the other children.” (A mother of two children).

Some parents mentioned that the parents of some of their children’s friends did not understand that there are many risks on a farm. The farming parents claimed that other parents sometimes send over their children as if the farm were a youth club. One parent said: “If I had known the scenario in advance we would probably have invited the parents of the children’s friends to come home to us on the farm and be informed about the risks. So they understand what the farm is about and that we cannot look after and take responsibility for their children at the same time as doing our work on the farm. We have tractors and horses that are very interesting and young people came and went all the time. The situation escalated and ended up in me forbidding my daughters to invite some of their friends and insisting that other children’s parents should be here if they want to go for a ride.” (A mother of three children).

#### Summary

The parents reported an increased risk of accidents with many children on the farm. Visiting friends create peer pressure, which makes children do things that they know are forbidden to get high status in front of other children. Despite this, many parents allowed their children to bring friends to visit their agricultural food industrial work environment, even if they had to go on with their own working tasks could not look after them properly.

### Farm risk education for children

The parents reported that they talk a lot with their children about where they are allowed to be on the farm. The parents saw this as a survival strategy. One father said: “We talk a lot with the children about where it is acceptable to be on the farm. It is a matter of survival.” (A father of two children).

A mother noted that they have special rules about how a child should react when they encounter a cow on their farm. She says: “We teach our children that it is important to talk to the cow and say “Hello little cow!” when they walk around in the barn, move around close to the cows or before they come near them, so the cow knows that something is going to happen. The cows then learn that this specific sound always is heard whenever they come close.” (A mother of three children).

Many of the parents claimed that their children learn about risks and safety by doing from an early age. One father said: “I think it is a question of training. If we are like a mother hen and shout “Look out, look out!!”, it will not be good. They must learn by themselves. Our task as parents is to make them independent. They have to test their ability by learn by their mistakes” (A father of three children).

Another parent said: “Sometimes I almost think that it will be good if some little incident happens. I mean so they understand that accidents really can happen. That they understand and learn from their own experience.” (A mother of three children).

Some of the parents mentioned that the environment on and around the farm was a safety risk but that children had to learn about it on their own. One parent said: “When our daughter was about three she started to follow us when we were in the barn on the other side of the main road. People who saw her when they drove through tell that she always looked both ways before crossing. She did this every morning, but what could we do about it? If we put up a fence she would probably climb over it. So, …… [he was quiet for a while]..... but luckily it has turned out well.” (A father of four children).

Many of the parents stated that it was important to talk and inform the children during the work and train them to have a safe working attitude. Some of the parents believed it was very important not to frighten the children when they informed and talked to them about the risky events and environments on the farm. This is because they do not want them to get the feeling that life on a farm is dangerous. These parents believed that it was the adults’ responsibility to make the environment as safe as possible. For some parents, it was also very important to be a good safety model to their children in their farm duties: “Both my husband and I try to be capable and examples for our children to model themselves on. We try not to do things with the engine on or so, we turn off the tractor before we attach or remove things and interact with the animals in a calm way so they not will be scared and so on.” (A mother of three children).

#### Summary

Many parents reported that their children learn to handle safety and avoid risks on the farm by trial and error. They stated that it was very important for children to participate in work on the farm so they built up safety awareness from the beginning, as a matter of life and death in many ways. However, the risk of this behaviour is that it could cost their children a life-long injury, handicap or early death. Agricultural parents seem to be unique in these opinions and behaviour. It is strange that parents in agriculture argue to learn their babies’ awareness about injuries and death by exposing them to their professional hazards.

## Discussion

Agriculture is the most damaging work environment, with most work-related fatal accidents and great risks to children living in this environment [1–18; 25;34;35;37]. The places on the farm parents perceived to be the most dangerous for their children were around traffic, animals, machines and drains, ponds, hay lofts and slurry tanks, but they pointed out that there were different risks in different age groups. Statistics from reported accidents show that traffic accidents are more common among younger children, while among adolescents, girls are most commonly injured by horse-related accidents and boys by machinery [14;25;37–42]. The parents in this study and the statistics both show that the risks differ with children’s age. Accidents involving younger children often occur because these children act unpredictably, and those involving older children because they are allowed to take part in more dangerous tasks [7;13;40;41]. Different types of ongoing activities and interventions are therefore needed to increase knowledge and awareness of risks and injury to children on farms [11;47–50]. However, reviews and evaluations of the effectiveness of different actions like policy solutions including formal and informal rules and regulations, risk education, modification of equipment and engineering controls, do not state any greater success [7;12–18;]. None of these actions seem to be effective enough to reduce child injury and death if the farmers do not voluntarily adopt the attitudes in their daily activities and tasks [17]. The most important thing to implement is therefore a changed risk attitude in the agricultural and rural community. How to make a risk behaviour change in agricultural is a clue and need to be multi-factual intervention activities including different risk areas [7–9]. Today there is lack of research on this topic, and more research is requested in the Nordic countries. Additionally, there are, as we know, no scientifically published evaluated Swedish interventions studies with farming parents aiming to reduce injuries related to children at farms.

The parents in this study noted that visiting friends create peer pressure, which makes children do things that they know are forbidden to get high status in front of other children. Peer pressure often exists in groups of young people and is not specific to farm children, but the working environment on farms is full of dangerous possibilities. Previous studies have also shown that young people who live, move around and play in this exposed environment run a greater risk of being injured [10], but that 25% of injured children are visitors [39]. Earlier studies stated that peer influence plays an important role in explaining risky behaviour during adolescence [51;52]. The adolescents also make riskier decisions in peer groups than they do alone. Additionally, youths focus more on the benefits, than the costs, of risky behaviour. Because of this, there can be an increased risk of injury when farm children have friends to visit, a fact that most of the parents interviewed mentioned. It is important to highlight increased risk of injury then many children come together in the preventive work to reduce the risk of injury to children on farms.

Some parents perhaps do not recognise the risks because they are blind to them, but some perhaps deny the threats in their children’s daily life situation because they consider the situation and conditions to be impossible to change [7;13;16;31–33]. The historical tradition of learning by experience at farms, suggests that early child experience in handling animals and machinery is a normal and essential part of learning to work at a farm [14;53]. However, this is a very dangerous way to learn an occupation. Additionally, the tradition of storytelling and macho challenging influences risk-taking behaviour and response to tragedy [14;54]. In work to decrease the number of accidents to children, it is encouraging to note that it is possible to change behaviour in a healthy direction by positive reinforcement and modelling [20–23]. However, the parents in this study also noted that society can decrease the safety risks by requiring safety devices or e.g. extra seats with safety belts as compulsory for tractors. To make things mandatory, e.g. as seatbelts in cars, have changed the attitude and almost everyone uses seatbelts in cars nowadays in Sweden. It could take some years to change an attitude on mandatory risk preventions, but to the new generations the new risk behaviours is the normal if they do not remember anything else.

Some of the parents also pointed out that males are more often involved in injuries and attributed this to men not taking farm safety questions as seriously as women and to risks and accidents on the farm being ignored if the work and family climate are too macho. Some mentioned specifically that women who did not grow up on a farm seem to think that almost everything on a farm is dangerous. Similarly, some fathers who did not grow up on a farm mentioned that their farm-bred wife had more liberal views of risks and children’s participation in daily farm chores. This account coincides with earlier reports [19;20;24;29–33], that risk-taking behaviour is modelled on influential others during childhood among both males and females.

Farms are often family businesses, which makes the situation between the parents’ work and the children’s safety much more complicated. Most of the parents in this study reported with pride that their children participate in work tasks on the farm. Family traditions, family relations, culture and feelings contribute to the economic situation and profit of the family business [16;21]. Family businesses often involve children and give them direct or indirect responsibility for business outcomes at an early age. A parent’s task is to protect their children. It is a very risky attitude to allow children to take part of occupational tasks with great risk of injury and accidents [13–16;41;44;55;56]. An attitude that perceive this behaviour as natural and normal do not realize the historical development of farms, i.e. from small scaled family farm to a modern and effective food production industry business. However, this behaviour could be a reflection of a business culture that values productivity over the human condition, i.e. the welfare of the next generation of children. Construction workers or industrial workers are not allowed to bring their children to their dangerous work site. Despite this, many farming parents’ attitude is still that it is normal behaviour to neglect the hazards their children are exposed to in the agricultural work environment [7;12–18]. This has to change to reduce individuals suffer. Additionally, there comes economic consequences with children’s injury. The productivity interest are probably not as large for parents who mourns their dead or injured children, and that could cause economic consequences for the family business. A children’s injury, handicap and death is also expensive to the society

The activities and tasks that the children in the present study were involved in depended on the children’s age. Children under the age of 10 did not have many tasks to do on their own, apart from feeding and looking after lambs or other baby animals and running errands. The parents did not seem to worry so much about adolescents aged about 15 and older, since they claimed that children of that age know the risks and dangers in the farm environment. Children aged between 10 and 15 appeared to be a borderline case. At that age the parents started to give them responsibility for doing some tasks on their own, e.g. driving tractors and milking cows. This is in violation of Swedish law, which states that children of this age are not mentally and physically ready for such tasks [26;27]. An evaluation of farm parents’ knowledge stated that they often do not know about the laws and regulations that restrict the child and teen work activities [55]. Another study stated that farm parents think that they them-self, and not the laws, should determine what work their children and teens should do [56]. According to earlier studies, parents normally overestimate their own children’s skills and abilities, although there can be great differences between children’s rates of development and maturity [42]. Older children are more likely to be injured than younger children [39]. Families and communities that suffer from a tragic injury or death of a child due to a farm- related incidence often lead to calls for strong protections and consequences in laws, lawsuits and public education, etc. To prevent and avoid accidents and bodily injuries among children in farming, it is important that the parents consider that the existing law is built on best practice and reliable experience. Therefore it is important to encourage parents to avoid giving their children dangerous tasks before they have the physical and physical maturity to cope with such tasks.

Many parents in this study reported that their children learn to handle safety and avoid risks on the farm by trial and error. Most of them believed that it was very important for children to participate in work on the farm so they built up safety awareness from the beginning, as a matter of life and death in many ways. However, to learn an occupation by trial and error in childhood does not seem to be more important in agriculture than in other occupations. This only seems to be a question of historical behaviour and attitudes, that threat to repeating a behaviour with lack of risk approach. Previous studies show that risk taking is often modelled on influential people in children’s immediate surroundings [19;20; 51;52]. The risk taking of supervisors, co-workers and individuals in youths immediate work environment are stated to be a strong predictor of youths’ risk-taking orientation at work [51;52]. If the aim is to teach children to avoid risks by participating in work on the farm, parents and other adults have a great responsibility to consider their own behaviour and act as a good model for the children. However, the best way to avoid children being injured or die in agricultural accident is to not allow them to participate in the work, in the same way children are not allowed to play or work in other dangerous workplaces. Future research and evaluations on interventions regarding optimal injury reducing multi-factorial actions in agriculture is needed [7–9].

## Conclusions

- Systematic work has going on for many years to protect children from bodily injury and it has reduced the number of children dying in accidents. However, there is much more to do, because there are still too many children being injured and dying every year in accidents on and around farms. The parents describe that there were some initiatives with educational approaches some years ago, but not anymore. Out from the result in this study and earlier studies [7–21; 31–33; 40–45; 49–56] occasional environmental and regulatory recommendations and educational approaches do not give a long time success. It is important to go on with this type of interventions and not only make them shorter projects that are forgotten after some years. It always come new hazards, additional to the old ones. It is therefore important to continuing educate children, new parents and new farmers about the hazards in the farm environment. It is also important to continuing with information campaigns, keep up the level of knowledge and remind parents and other adults about things that they sometimes already know, but neglect. This is very important in decreasing the number of children at risk also in the future and in the long time perspective.
- Mandatory safety standards take some years to implement, and it is important to realize that no activities aimed to reduce child injury and death seems to work if the farmers do not voluntarily adopt them in their daily work. Therefore, it is important to perform a cultural change of the risk attitude in the entire agricultural and rural community. It is probably easier to be more familiar with safety rules, as seatbelts in tractors and ROPS, if people grew up with them. This could also be an explanation to that the attitude to accept safety regulations and parents’ desires of more seatbelts in tractors could be different in different countries and cultures. It is also important that farm children and young people in the most exposed groups are given priority in this work.
- At some farms business seem to be favouring before a good work environment and risk behaviour. However, good risk behaviours and providing a good work environment favouring business in the long time. Because a parents with an injured or dead children could probably not produce as good due to their grief, as a parent with a healthy child. Children and adolescent safety is therefore also economically good for the business and for the society.
- Additional is it important to realise the societies roll in the promotion of child safety. The entire responsibility for children’s safety on farms cannot be laid only on the parents’ shoulders. Society has an important role to play in supporting the parents with preventive measures, engineering controls and in reducing the risks for every age group on farms. However, if the society and farming parents really want to reduce injury and death of children it is time to realize that farming is not a child’s job. Maybe it is time to make the historical prevention step and separate children and family living from the agricultural food production industry in order to protect them, i.e. like it is in all other occupations with dangerous work environments.
- Further research and evaluation of intervention is needed to prevent injury, change risk attitude and promote a healthy safety behaviour in agriculture. A future research is also to study if this is as common in other dangerous occupations as in agriculture to learn babies’ awareness about injuries and death by exposing them to the parents’ professional hazards.

## References

[1] Nordic Council of Ministers. Fatal Occupational Accidents in the Nordic Countries 2003–2008, Tema Nord 2011: 501. Copenhagen, Denmark, 2011.

[2] The Swedish Work Environment Authority. Occupational injuries 2011. Report 2012:2. Stockholm: Statistic Sweden; 2012.

[3] Swedish Board of Agriculture. Agricultural statistics 2008 Stockholm, Sweden: Statistics Sweden; 2008. (In Swedish).

[4] Nilsson K, Pinzke S, Lundqvist P. Occupational injuries to senior farmers in Sweden. J Agr Saf Health. 2010; 16(1): 19. 20222268 10.13031/2013.29246

[5] McCall BP, Horwitz IB, Carr BS. Adolescent Occupational Injuries and Workplace Risks: An Analysis of Oregon Worker’s Compensation Date 1990–1997. J Adolescent Health. 2007; 4: 248. 10.1016/j.jadohealth.2007.02.004 17707294

[6] The Swedish Work Environment Authority. Occupational Accidents and Work related Diseases 2007, Report 2008:2. Stockholm: Statistic Sweden; 2008. (in Swedish)

[7] Hartling L, Brison RJ, Crumley ET, Klassen TP, Pickett W. A Systematic Review of Interventions to Prevent Childhood Farm Injuries. Pediatrics 2004;114(4): 483–496. 10.1542/peds.2003-1038-L 15466075

[8] Rautianinen R. Lehtola MM. Day LM. Schonsteine: Suutairinen J. Salminen S. Verbeek JH. Interventions for preventing injuries in the agricultural industry. Cochrane Database of Systematic Reviews, 2008, Issue 1. Art.No.:CD006398. 10.1002/14651858.CD006398.pub2 PMC12433932 18254102

[9] Nilsson K. Interventions to reduce injuries among older workers in agriculture: A review of evaluated intervention projects. WORK: A Journal of Prevention, Assessment, and Rehabilitation, 2016, On line first (Accepted 20151202)

[10] Swedish Ministry of Health and Social Affairs. Social differences in hazards among children and youth SOU 2002:68. Government Offices of Sweden; 2002. (in Swedish)

[11] Lee BC, Gallagher SS, Liebman AK, Miller ME, and Marlenga B, (Eds.) Blueprint for Protecting Children in Agriculture: The 2012 National Action Plan. Marshfield, WI: Marshfield Clinic; 2012 https://www3.marshfieldclinic.org/proxy///mcrf-centers-nfmc-nccrahs-2012_blueprint_for_child_ag_inj_prev.1.pdf 10.1080/1059924X.2012.660437 22490023

[12] Hard DL. Partering Strategies for Childhood Agricultural Satety and Health. Journal of Agromedicien, 2012;17:225–231. 10.1080/1059924X.2012.658341 PMC4678869 22490034

[13] Miller ME, Historical Background of the Child Labor Regulations: Strengths and Limitations of the Agricultural Hazardous Occupations Orders. Journal of Agromedicine, 2012;17:163–185. 10.1080/1059924X.2012.660434 22490029

[14] Wright S, Marlenga B, Lee B. Childhood Agricultural Injuries: An Update for Clinicians. Curr probl Pediatr Adolesc Health Care, 2013;43:20–44. 10.1016/j.cppeds.2012.08.002 23395394

[15] Morrongiello BA, Zdzieborski D, Stewart J. Supervision of Children in Agricultureal Settings: Implications for Injury Risk and Prevention. Journal of Agromedicine, 2012;17:149–162. 10.1080/1059924X.2012.655127 22490028

[16] Schwebel DC, Pickett W. The Role of Child and Adolescent Development in the Occurrence of Agricultural Injuries: An Illustration Using Tractor-Related Injuries. Journal of Agromedicine, 2012;17:214–224. 10.1080/1059924X.2012.655120 22490033

[17] Lehtola MM, Rautianen RH, Day LM, Schonstein E, Suutarinen J, Salminen S, et al Effectiveness of interventions in preventing injuries in agriculture–a systematic review and meta-analysis. Scandinavian Journal of Work, Environment & Health, 2008:34(5):327–337. 10.5271/sjweh.1279 18853066

[18] Gallagher AA. Characteristics of Evaluated Childhood Agricultureal Safety Interventions. Journal of Agromedicine, 2012;17:109–126. 10.1080/1059924X.2012.664033 22490025

[19] Skinner BF. About Behaviorism. New York: Alfred A. Knopf; 1974.

[20] Bandura A, Walters RH. Social Learning and Personality Development New York: Holt, Rinehart and Winston, INC; 1963.

[21] Bandura A. Principles of Behavior Modification London: Holt, Rinehart and Winston. INC; 1969.

[22] Glanz K, Marcus Lewis F, Rimer BK. Health Behavior and Health Education Teory, Research and Practice. San Francisco: Jossey-Bass Publishers; 1997.

[23] Naidoo J, Wills J. Health Promotion Foundations for Practice. London: Baillière Tindall; 2000).

[24] Sorensen J. Social Marketing for Injury Prevention Changing risk perceptions and safety-related behaviors among New York farmers. Umeå University; 2009.

[25] Swedish Ministry of Health and Social Affairs. Children injury in Sweden 1987–2000. SOU 2002:99. Government Offices of Sweden; 2002. (in Swedish)

[26] The Work Environment Act (1977:1160) The Swedish Legislation.

[27] AFS 2012:3. Young people at work. Stockholm: The Swedish Work Environment Authority; 2012. http://www.av.se/dokument/afs/afs2012_03.pdf

[28] Pinzke S, Nilsson K, Lundqvist P. Farm tractors on Swedish public roads—Age-related perspectives on police reported incidents and injuries. Work 2014;49(1):39–49 10.3233/WOR-131767 24284664

[29] Hall A. Strategizing in the context of genuine relations An interpretative study of strategic renewal through family interaction. Jönköping: International Business School. Högskolan Jönköping; 2003.

[30] Farmsafe Australia. Child Safety on Farms A framework for a national strategy. Moree: Farmsafe Australia; 1999.

[31] Cullberg J. Crisis and Development. Stockholm: Natur & Kultur; 2001. (in Swedish)

[32] Zerubavel E. The Elephant in the Room: Silence and Denial in Everyday Life. Oxford: University Press; 2006.

[33] Koskela H, Pain R. Revisiting fear and place: woman’s fear of attack and the built environment. Geoforum 2000;31:269–280.

[34] Pinzke S, Lundqvist P. Work accidents in forestry and agriculture 2004 Alnarp: The Swedish University of Agricultural Sciences; 2006. (In Swedish)

[35] Östberg V. The social patterning of child mortality: the importance of social class, gender, family structure, immigrant status and population density. Sociology of Health & Illness 1997:19 (4)415–435.

[36] Swedish Ministry of Health and Social Affairs. From Children’s Risk of Accident to Children’s Right of Safety and Development. SOU 2003:127. Stockholm: The Delegation of Children’s Safety Government Offices of Sweden; 2003. (in Swedish)

[37] Benfalk C, Ringmark A. Children Accidents on Agricultural Company. Uppsala: Swedish Institute of Agricultural and Environmental Engineering; 2007. (in Swedish).

[38] The Norwegian Farmers´ Association for Occupational Health and Safety. Safer farm–holding and planning Children and youth in agriculture. The Norwegian Farmers´ Association for Occupational Health and Safety; 2000. (in Norwegian)

[39] National Agricultural Statics Service (NASS) 2001 Childhood Agricultural–Related Injuries Fact Finders for Agriculture. Washington D.C.: United States Department of Agriculture; 2004.

[40] Morrongiello BA, Marlenga B, Berg R, Linneman J, Pickett W. A new approach to understand pediatric farm injuries. Social Science & Medicine 2007;65:1364–1371. 17583403 10.1016/j.socscimed.2007.05.013

[41] Morrongiello BA, Pickett W, Berg RL, Linneman JG, Brison R, J, Marlenga B. Adult supervision and pediatric injuries in the agricultural worksite. Accident Analysis and Prevention 2007;40:1149–1156. 10.1016/j.aap.2007.12.007 18460383

[42] Lueder R, Berg Rice VJ. Ergonomics for Children Designing products and places for toddles to teens. New York: Taylor & Francis; 2008.

[43] Thelin A. Fatalities in farming and forestry: an examination of registry information used in the Swedsih national statistics. Journal of Agricultural Safety and Health 2002;8:289–295. 12363180 10.13031/2013.9053

[44] Marlenga B., Pickett W, Berg R, Murphy D. Operational characteristics of tractor driven by children on North American farms. Journal of Agricultural Safety and Health 2004;10:17–25. 15017802 10.13031/2013.15671

[45] Hagström C, Marander-Ekelund L. Question list as sours and method Lund: Studentlitteratur; 2005 (in Swedish)

[46] Kvale S. The qualitative research interview Lund: Studentlitteratur; 1997. (in Swedish)

[47] CIPA (Child Injury Prevention Alliance). Keeping kids safe on farms. A guide for farming families; 2012. www.childinjurypreventionalliance.com

[48] Esser N, Heiberger S., Lee B. (Eds.) Creating Safe Play Areas on Farms. Marshfield, WI: Marshfield Clinic; 2003 http://www3.marshfieldclinic.org/proxy/MCRF-Centers-NFMC-NCCRAHS-resources-SafePlayBooklet2012.1.pdf

[49] Säkert Bondförnuft, Child safety on the farm; 2013. www.sakertbondfornuft.se

[50] The Australian Centre for Agricultural Health and Safety. Child Safety on Farms. Number 7: Agricultural Health and Safety Guidance Note Series. Moree. Australia; 2004.

[51] Westaby J, Krister J. Lowe Risk-Taking Orientation and Injury Among Youth Workers: Examining the Social Influence of Supervisors, Coworkers, and Parents. Journal of Applied Psychology Copyright 2005 by the American Psychological Association, 2005; 90(5)1027–1035. 10.1037/0021-9010.90.5.1027 16162075

[52] Gardner M, Steinberg L. Peer Influence on Risk Taking, Risk Preference, and Risky Decision Making in Adolescence and Adulthood: An Experimental Study. American Psychological Association, 2005;41(4)625–635. 10.1037/0012-1649.41.4.625 16060809

[53] Sanderson LL, Dukeshire SR, Rangel C, Garbes R. The farm apprentice: agricultural college students recollections of learning 10.13031/2013.34835 21180348

[54] Robertson SM, Murphy DJ, Davies LA. Social and emotional impacts of farmwork injuries: an exploratory study. Journal of Rural Health, 2006;22:26–35. 16441333 10.1111/j.1748-0361.2006.00001.x

[55] Runyan CW, Vladutiu CJ, Schulman MD, Rauscher KJ. Parental involvement with their working teens. Journal of Adolescents Health, 2011;49:84–86. 10.1016/j.jadohealth.2010.09.024 21700162

[56] Runyan CW, Schulman M, Dal Santo J, Bowling JM, Agans R. Attitudes and beliffes about adolscents work and workplace safety among parents of working adolescents. Journal of Adolescents Health, 2009;44:349–355. 10.1016/j.jadohealth.2008.08.009 19306793

[57] Nilsson K. Föräldrars attityder till barn och ungdomars uppväxtmiljö, risker och olyckstillbud på lantbruk. [Parents' attitudes to children and adolescents living environment, risks and injuries on farms] Swedish University of Agricultural Sciences, Alnarp. (in Swedish.)

